# Recent trends in Bi-based nanomaterials: challenges, fabrication, enhancement techniques, and environmental applications

**DOI:** 10.3762/bjnano.13.109

**Published:** 2022-11-11

**Authors:** Vishal Dutta, Ankush Chauhan, Ritesh Verma, C Gopalkrishnan, Van-Huy Nguyen

**Affiliations:** 1 School of Advanced Chemical Sciences, Shoolini University, Solan, Himachal Pradesh 173212, India; 2 Chettinad Hospital and Research Institute, Chettinad Academy of Research and Education (CARE), Chengalpattu district, Kelambakkam, Tamil Nadu, 603103, India; 3 University Centre for Research and Development, Chandigarh University, 140413, India; 4 Department of Physics and Nanotechnology, SRM Institute of Science and Technology, Tamil Nadu, 603203, India

**Keywords:** bismuth-based nanomaterials, environmental remediation, heterojunction formation, photocatalysis

## Abstract

One of the most enticing approaches to environmental restoration and energy conversion is photocatalysis powered by solar light. Traditional photocatalysts have limited practical uses due to inadequate light absorption, charge separation, and unknown reaction mechanisms. Discovering new visible-light photocatalysts and investigating their modification is crucial in photocatalysis. Bi-based photocatalytic nanomaterials have gotten much interest as they exhibit distinctive geometric shapes, flexible electronic structures, and good photocatalytic performance under visible light. They can be employed as stand-alone photocatalysts for pollution control and energy production, but they do not have optimum efficacy. As a result, their photocatalytic effectiveness has been significantly improved in the recent decades. Numerous newly created concepts and methodologies have brought significant progress in defining the fundamental features of photocatalysts, upgrading the photocatalytic ability, and understanding essential reactions of the photocatalytic process. This paper provides insights into the characteristics of Bi-based photocatalysts, making them a promising future nanomaterial for environmental remediation. The current review discusses the fabrication techniques and enhancement in Bi-based semiconductor photocatalysts. Various environmental applications, such as H_2_ generation and elimination of water pollutants, are also discussed in terms of semiconductor photocatalysis. Future developments will be guided by the uses, issues, and possibilities of Bi-based photocatalysts.

## Introduction

Nanomaterials photocatalysis is a “green” integrative technique that combines physics, chemistry, and materials science with chemical engineering to catalyze chemical processes and transform constantly recoverable solar energy into productive chemical energy [1]. Various semiconductor nanoparticles have been used as effective photocatalysts in essential photocatalytic applications such as wastewater treatment, water dissociation, and energy conversion/storage due to their reactivity, surface area, and advantageous features compared to their bulk counterparts [2–4]. In recent years, many efforts have increased the photocatalytic performance. However, the relative photocatalytic performance is still deficient, and it does not fulfil the criteria for the practical implementation of photocatalysis techniques. Among many approaches, attention has been paid to altering and modifying the properties of photocatalytic materials [5–6]. Environmental treatment and energy conversion using photocatalytic technology have shown to be cost-effective and environmentally beneficial alternatives [7]. The choice of the photocatalysts is one of the most important steps in attaining high performance in photocatalysis. Semiconductors with bandgaps greater than 3 eV are called wide-bandgap photocatalysts. These semiconductors include oxides (e.g., TiO_2_, Bi_2_O_3_, Bi_2_WO_6_, and SrTiO_3_), sulfates (e.g., MoS_2_ and Bi_2_S_3_), selenides (e.g., MoSe_2_ and CdSe), and phosphates (e.g., Ag_3_PO_4_) [8–15].

The bandgap of photocatalysts sensitive to visible light is smaller than 3 eV. Wide-bandgap photocatalysts can only be stimulated by ultraviolet light, which makes up less than 5% of the absorbed solar radiation. Hence, developing photocatalysts that react to visible light is essential for photocatalysis since 43% of the total energy from the sun belongs to the visible spectrum [1,16]. Bi-based nanomaterials are photocatalysts that respond to visible light and have adequate bandgaps and performance. Bi_2_MO_6_ (M = Mo, W), (BiO)_2_CO_3_, Bi_2_S_3_, BiOX (X = I, Br, Cl), BiPO_4_, BiVO_4_, Bi_2_O_3_, and other Bi-based nanomaterials have been designed and examined for photocatalysis. The vast majority of these compounds have a layered structure, which causes an internal electric field (IEF) between the layers. This electric field allows photogenerated charge carriers to be separated and moved effectively [17–21].

**Figure 1:**
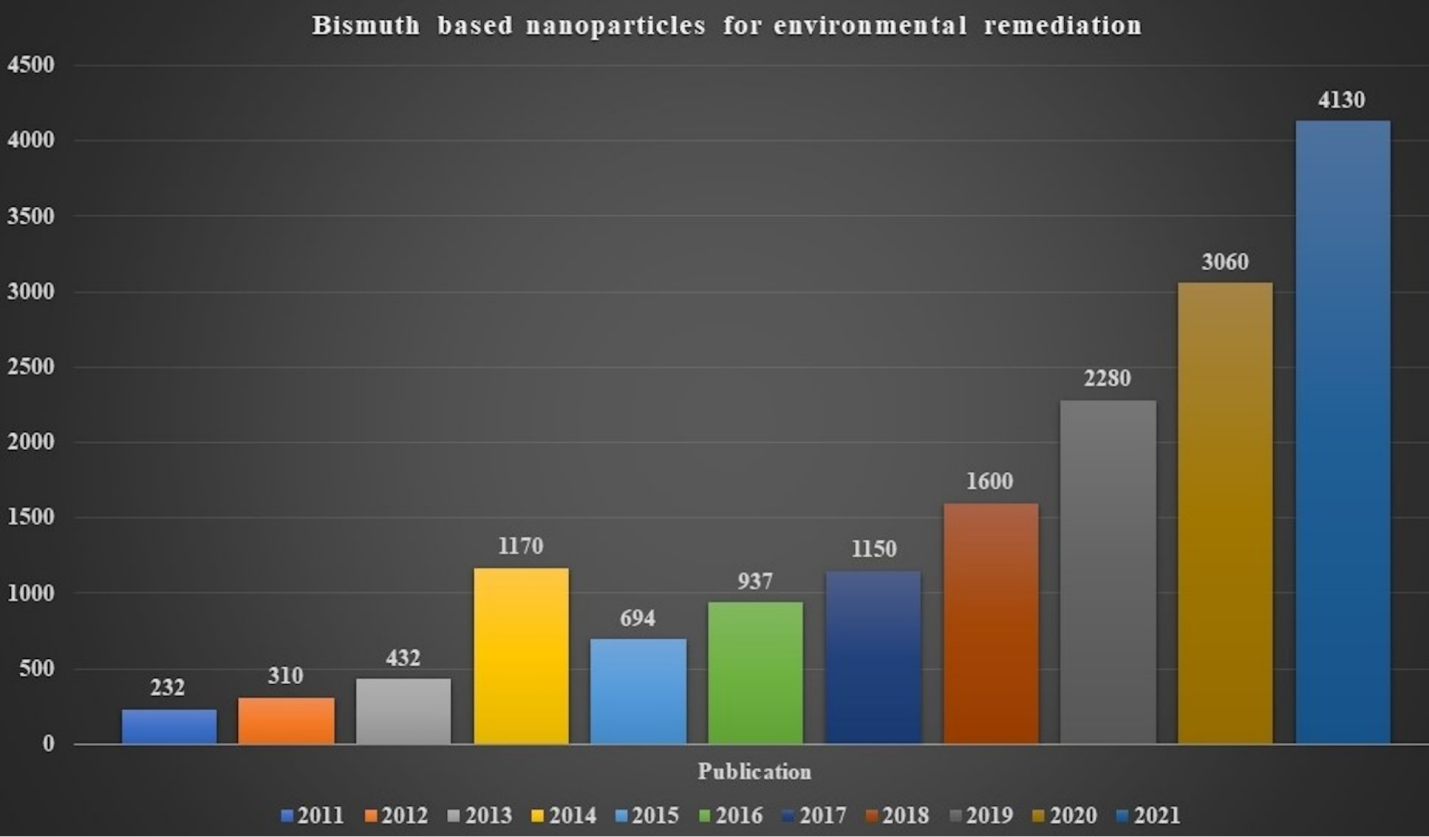
Reported publications from 2011 to 2021 were searched on 6th June 2022 using the keyword “Bismuth-based nanoparticles for environmental remediation”. Data was extracted from Web of Science, Clarivate Analytics.

A range of visible-light-active Bi-based photocatalysts has lately raised curiosity among semiconductor photocatalysts. Bi^3+^ has a higher stability than Bi^5+^. The earlier compounds have been examined more thoroughly than the latter. The overlap of O 2p and Bi 6s orbitals in the valence band (VB) of Bi^3+^-containing compounds improves photogenerated charge mobility and, hence, improves photocatalytic activity [22–23]. Furthermore, except for BiOF, BiOCl, and (BiO)_2_CO_3_, most Bi-based compounds have bandgaps that may be stimulated by visible light. As a result, much interest in environmental cleanup and energy conversion research has been sparked by Bi compounds [24]. Because of their advanced photocatalytic process, more and more publications on synthesizing and applying a semiconductor photocatalyst have been published in recent years. A survey on bismuth-based nanocomposites with the search keywords "Bismuth-based nanoparticles for environmental remediation" from 2011 to 2021 yields roughly 15,995 articles. This data illustrates the interest of the scientific community in environmental cleanup using bismuth-based nanoparticles (Figure 1). In recent years, an abundance of Bi-based photocatalysts has been reported.

The most commonly used Bi-based photocatalysts include metallic Bi, Bi-based binary oxides, Bi-based oxyhalides, Bi-based multicomponent oxides, and binary Bi sulfides. Bismuth oxyhalides are indirect bandgap semiconductors in which photogenerated electrons and holes rarely recombine. BiOX is an excellent photocatalyst, and it is widely applied due to its small bandgap and high electron density, which are easily adjustable by changing the type of halogen used. The activation of otherwise inert CO_2_ and H_2_O molecules is greatly aided by the ease with which photoinduced oxygen vacancies (OVs) are produced on the surface [25]. The excellent photocatalytic performance can be attributed to the layered crystal structures and small bandgap energies. Many persistent organic contaminants can be degraded at room temperature through the oxidizing power of VB holes in bismuth oxyhalides [26]. BiOCl, BOI, BiOBr, and composites made from them have been widely reported due to their excellent photocatalytic properties [27–29]. However, the photocatalytic effectiveness of those semiconductors is inadequate for practical environmental and energy conservation applications because of substantial electron–hole recombination and a low capacity for the absorption of visible light. Numerous attempts have been made, with an emphasis on doping, the creation of heterojunctions, crystal plane management, and defect development, to enhance the photocatalytic efficacy of pristine Bi-based photocatalysts [1,30]. The photocatalytic processes of Bi-based photocatalytic applications have received little attention in environmental remediation and energy conversion. The rapidity with which this vital subject is advancing necessitates a thorough examination of recent breakthroughs regarding Bi-based photocatalysts. Consequently, in this work, Bi-based semiconductor photocatalysts and their manufacturing methods are discussed to make use of these photocatalysts in eco-friendly applications on a large scale. Bi-based nanomaterials as semiconductor photocatalysts are one of the study’s primary goals, as is the use of Bi-based nanomaterials for wastewater treatment, hydrogen generation, and photocatalytic degradation. Fabrication methods, reliability analogies, and future challenges of photocatalysts derived from bismuth-based nanomaterials are also discussed. There are many review reports on synthesis and enhancement techniques of Bi-based photocatalysts and the application of these photocatalysts in hydrogen generation, CO_2_ reduction, and water purification [31–35]. However, the present report focuses on understanding the role of different Bi-based photocatalysts concerning their synthesis method and enhancement. The mechanism of photocatalysts for different applications has been described for the type or nature of the photocatalyst. Hopefully, this work will help researchers understand how Bi-based nanomaterial photocatalysts may be employed in different environmental remediation systems by understanding the properties of Bi-based nanomaterials photocatalysts.

## Review

### Photocatalysis

#### Photocatalysis and its challenges

Photocatalysis can transform solar energy into storable chemical energy. Because of its minimal energy intake and carbon footprint, it is eco-friendly and promising. Two examples are the conversion of CO_2_ to hydrocarbons and water splitting to H_2_ and O_2_ [36–37]. Also, it is essential in domains including pollution degradation, antibiotic treatment, and sterilization [38]. The term “advanced oxidation processes” has become more common recently. In this process, many oxidizing agents (∙OH) are created. Electron–hole pairs are formed in AOPs when the VB electrons of semiconductor photocatalysts are driven into the conduction band (CB) through visible light [39]. The holes in the valence band of the catalyst split water to hydroxyl radicals (^•^OH). Electrons in the CB of a semiconductor photocatalyst can generate the superoxide anion (^•^O_2_^−^) when they interact with oxygen molecules. During the photocatalytic oxidative degradation, the most notable oxidizing species are ^•^OH, photogenerated holes, and ^•^O_2_^−^. These species are responsible for the photodegradation of organic and inorganic contaminants in wastewater [40]. To date, it is widely understood that the main limitation of photocatalysts is their low photocatalytic efficiency. The reason is that photogenerated electrons and holes recombine quickly [41]. We use an analogy for a more straightforward comprehension of the recombination timeframe. The force of gravity ensures that any item thrown into the air will return to the Earth below within a few seconds. After being exposed to light, electrons in a single photocatalyst undergo a transition akin to an item thrown into the air. This transition takes place from the VB to the CB [42]. After that, the very powerful Columbic force among photogenerated electrons and holes pulls them together, enabling recombination within a few picoseconds to nanoseconds in bulk or on the photocatalyst surface. It is possible to have a better understanding of the timescale by contrasting the calculation of the gravitational force with the computation of the Coulomb force. Because the gravitational constant (6.67 × 10^−11^ N·m^2^·kg^−2^) is significantly smaller than the Coulomb constant (8.99 × 10^9^ N·m^2^·C^−2^), the recombination of the photogenerated electron and hole pairs takes significantly less time than the fall of the object. Within a single photocatalyst, photogenerated electrons and holes cannot withstand the tremendous force, which results in rapid recombination [43]. In addition to a low rate of recombination, other essential qualities of a superior photocatalyst include broad sunlight absorption and enough redox capacity. A small bandgap is desirable regarding a broad light absorption band. However, when redox ability is considered, the catalyst should have a high CB position and a low VB position, resulting in a large bandgap. These two prerequisites are not compatible. As a direct consequence of this, heterojunctions are produced. To create heterojunction photocatalysts, two semiconductor photocatalysts are combined [44–45]. Consequently, researchers focus their attention mainly on heterojunction photocatalysts.

#### Promising Bi-based nanomaterials

**Figure 2:**
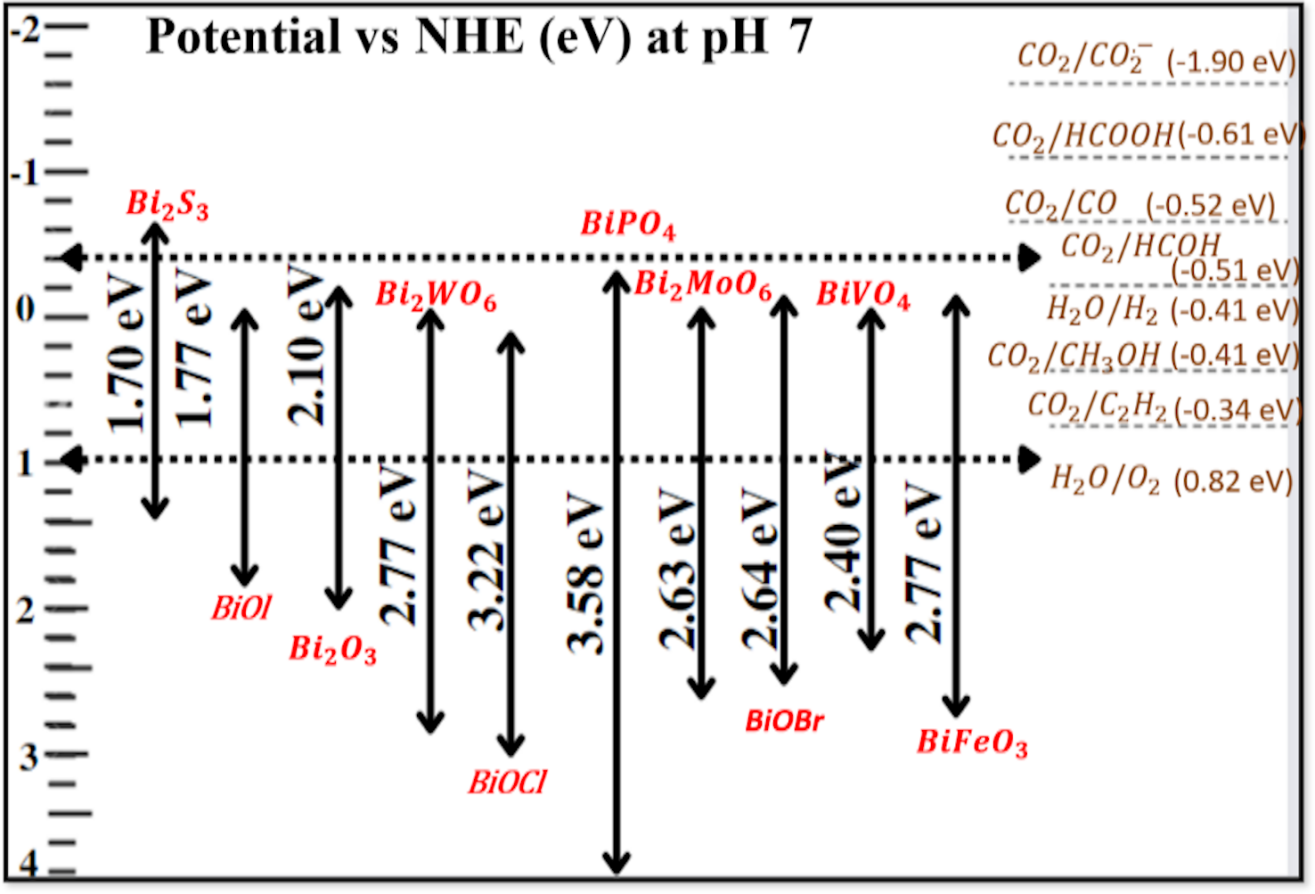
Bi-based photocatalysts exhibit substantial oxidative capabilities for various redox processes.

The overwhelming number of Bi-based semiconductors utilized in photocatalysis also have a distinctive layered structure and a bandgap of less than 3.0 eV. The connections between the layers are just van der Waals forces, which are weak [46]. Metal oxides such as TiO_2_ only have the O 2p orbital in their VB. In contrast , Bi-based oxide materials have an electronic structure in which O 2p and Bi 6s orbitals are paired in the VB. The bandgap of the semiconductor may be reduced to 3.0 eV thanks to the significant charge carrier dispersion provided by hybrid orbitals involving the Bi 6s orbital, as seen in Figure 2. Photoinduced electron–hole separation and charge carrier transfer in Bi-based materials are facilitated by a unique layered structure that creates an IEF. A magnetic field is generated between layers of Bi-based materials [47]. Many researchers have revealed that Bi-based nanomaterials have an adequate photocatalytic capacity for pollution remediation, water splitting, and the elimination of volatile organic compounds. Bi-based photocatalysts have substantial oxidative capabilities, as illustrated in Figure 2, since their VB potential is much higher than the oxidation potential of H_2_O, that is, 0.82 V vs NHE. Unfortunately, due to inadequate CB potential energy, most reduction processes, such as CO_2_ reduction, N_2_ fixation, and H_2_ creation, cannot be catalyzed with Bi-based nanomaterials. However, a few Bi-based semiconductor photocatalysts, such as Bi_2_S_3_, have a more substantial negative CB potential, making reduction reactions possible [34,48–49]. However, in real applications, the usefulness of Bi_2_S_3_ is limited because of the quick recombination of electrons and holes. New research has shown that it is possible to generate H_2_ or reduce CO_2_ by carefully controlling the production procedure of ultrathin photocatalysts based on Bi. Compared to Bi_2_WO_6_ formed using a solid-state reaction, single-unit-cell layers of Bi_2_WO_6_, synthesized using a hydrothermal process, offered three times greater CO_2_ adsorption and enhanced light absorption. The enhanced properties were reflected in the photocatalytic activity, resulting in a rate of methanol synthesis of 75 mol·g^−1^·h^−1^, which was much greater than the rate produced by the unaltered Bi_2_WO_6_. As a result of the decrease in dimensionality, it was projected that there would be a significant confinement effect and enlarged bandgap energy. Additionally, an upshift of the CB and a downshift of the VB were also measured [48,50]. The preparation process can be used to modify the electrical structure of Bi-based semiconductors. Most Bi-based semiconductors, especially oxide semiconductors such as Bi_2_O_3_, BiVO_4_, and Bi_2_WO_6_, have n-type properties with electrons as the majority carrier. Recent research suggests that the synthesis route can shift the conductivity types of Bi-based materials [45,51].

### Fabrication routes

Synthesis approaches affect the topological size and size distribution of semiconductor photocatalysts, substantially influencing adsorption characteristics and photocatalytic efficacy. As well as affecting the environment, synthesis size, and cost, the fabrication method also affects manufacturing safety considerations [52]. Bi-based photocatalysts may currently be synthesized using various methods, the most common of which are hydrothermal synthesis, solid-state reaction, and design and optimization.

Today, NaBiO_3_·2H_2_O, Bi_2_O_3_, Bi(NO_3_)_3_·5H_2_O, BiCl_3_, and Bi are the most common Bi-based semiconductor photocatalysts. The hydrothermal process is one of the most frequent and widely utilized synthesis methods. Morphology, facets, size, surface flaws, and dimensionality of Bi-based photocatalysts are all susceptible to change when the synthesis conditions are altered. Generally, the hydrothermal technique yield a higher quality, and the nanoparticles that result from this process are more suited for specific applications than photocatalysts manufactured using dry methods [53]. However, the hydrothermal approach has a noticeable limitation in output yield. The usage of specific autoclaves has resulted in a long manufacturing time and the batch nature of production. For the hydrothermal technique, there is a danger of nanoparticle leakage, primarily in water, and the risk of toxic solvent emissions. Su et al. [54] reported the first hydrothermal fabrication of Bi_5_O_7_Br. Bi_5_O_7_Br rods of 50 μm length and 2 μm width were obtained. They found that Bi_5_O_7_Br effectively converts molecular oxygen to superoxide radicals and hydroxyl radicals in visible light. Under UV–vis irradiation, Bi_5_O_7_Br showed a higher photocatalytic activity in the degradation of rhodamine B (RhB) dye than BiOBr. The addition of Bi_5_O_7_Br photocatalysis to the Bi–O–X photocatalytic system improved the system. In this work, they found that the RhB elimination percentage over Bi_5_O_7_Br is 85% after 120 min of UV–visible-light irradiation, and the reaction rate constant was measured as 1.496 h^−1^·m^−2^. In contrast, the reaction rate constant for BiOBr was found to be only 0.154 h^−1^·m^−2^. Additionally, the hydrothermally fabricated Bi_5_O_7_Br has also excellent stability when exposed to light in aqueous environments. The photocatalytic performance was not much decreased after eight cycles. Recently, Lin et al. fabricated a monoclinic BiVO_4_ photocatalyst via a surfactant-free hydrothermal technique [55]. According to the results, the pH value has a significant impact on shape, surface area, particle size, and V–O bond length. The grain size was reduced when the pH value was raised, and the crystal structure became more closely stacked. Under visible-light irradiation, the RhB photodegradation efficiency of the coralloid particles produced at pH 7 was about four times greater than that of the sample synthesized at pH 0.5. The increased photocatalytic activity was caused by several factors, such as a synergistic effect of highly exposed (010) grain facets, an increase in the overlap between Bi 6s and O 2p orbitals, a reduced charge transfer route, and strange floating features. In another approach, Huang et al. reported a one-step hydrothermal synthesis of a BiIO_4_/Bi_2_MoO_6_ hybrid photocatalyst for photodegradation of RhB dye [56]. Under visible-light irradiation (λ > 420 nm), photoelectrochemical studies demonstrated that the RhB photodegradation effectiveness and photocurrent density of the BiIO_4_/Bi_2_MoO_6_ hybrid composite is much higher than that of the pure components. Because of the successful construction of the BiIO_4_/Bi_2_MoO_6_ hybrid, the photocatalytic activity was found to be significantly increased. This improvement was ascribed to the efficient interfacial charge transfer obtained as a consequence of the process. According to the active species trapping study, the photocatalysis process is significantly aided by the presence of photogenerated holes.

It has been shown that the hydrothermal method of fabricating Bi-based semiconductor photocatalysts offers some significant benefits. Nanostructured materials have several advantages, including a more adaptable area for more detailed reconstruction, confinement effects, superior mechanical stability, and large surface area, which make them excellent for photocatalysis. The progress of synthesis processes allows one to change the physical properties as needed.

In order to create economically viable Bi-based photocatalysts, substantial amounts of water are required. Solid-state reaction methods that do not require water are suitable for large-scale synthesis. However, in solid-state reaction methods, there is the risk of a release of nanoparticles into the air; as a result, they are not entirely eco-friendly [57]. He et al. reported the fabrication of a Bi_4_NbO_8_Br photocatalyst via solid-state synthesis [58]. They found that the temperature used during calcination plays a vital role. A higher calcination temperature results in a more crystalline morphology with more active sites for photocatalytic activity. The fabricated specimens were utilized for the photodegradation of RhB under visible light. The sample calcinated at 750 °C revealed the highest photocatalytic performance. Hamza et al. fabricated Bi_2_(CrO_4_)_3_ nanoparticles via a facile precipitation technique [59]. The photocatalytic activity of the Bi_2_(CrO_4_)_3_ nanoparticles was studied under UV, AM 1.5, and visible-light irradiation, and acceptable rates of 522.44, 174.15, and 88.24 μmol·g^−1^·h^−1^, respectively, were reached under these conditions. These rates outperform those of other similar Bi-based semiconductor photocatalysts reported in the literature.

To synthesize Bi-based photocatalysts with high anisotropy, hollow structures, or crystalline multidimensional forms, the template technique is one of several synthesis approaches. Direct manufacturing processes make this almost impossible. Therefore, using templates is a great workaround [60]. Template methods may be characterized either as a “hard templates”, “soft templates”, or “self-templates”. The high cost of template methods results from the lengthy process of creating and removing templates. Also, environmental aspects need to be considered as removing templates such as SiO_2_ requires using very corrosive acids or bases [61]. Numerous studies have focused on choosing templates for making nanostructures of functional materials. However, templates were chosen rather based on utility than on cost. The self-template technology does not require additional templates, which leads to less expensive production and increased efficiency. This makes the approach more realistic for practical applications [62]. A very good micro-/nanoscale hierarchical Bi_7_O_9_I_3_/NTC photocatalyst was created in a one-step, easy, and environmentally friendly way by Hou et al., who used an in situ ion exchange–recrystallization approach [63]. The used buffer provided a relatively stable environment for producing regular structures. The aqueous NH_3_ solution provided OH^−^ ions for the successful exchange of I^−^, and the result was the synthesis of Bi_7_O_9_I_3_. The Bi_7_O_9_I_3_/NTC has evenly distributed Bi_7_O_9_I_3_ nanostructures in the shape of lanterns formed of extremely thin nanosheets with a thickness of less than 10 nm on both the surface and the inside. When exposed to visible light, Bi_7_O_9_I_3_/NTC displayed higher photocatalytic activity owing to the synergistic effect of the micro-/nanoscale hierarchical structure, low iodine content, and well-contacted interface. 93.5% methyl orange (MO) and 96.6% RhB were eliminated from solution during in two hours, suggesting a greater photocatalytic effectiveness than that of pure BiOI. To deposit metallic Bi on Bi_2_WO_6_ nanosheets, an in situ reduction approach using NaBH_4_ as the reducing agent was used [64]. Compared to pure Bi_2_WO_6_, Bi-coated Bi_2_WO_6_ absorbs more visible light, is more sensitive to photocurrent, and has a lower electrochemical impedance rate. This is because of surface plasmon resonances (SPRs) and the electron transport capabilities of Bi. The photocatalytic activity for the breakdown of phenol was significantly improved, compared to pristine Bi_2_WO_6_ under visible light. Xiao and colleagues have shown a straightforward synthesis approach for fabricating Bi_2_WO_6_ nanosheet rods [65]. They discovered that the hydrolysis of the precursor Bi(NO_3_)_3_ may quickly result in the formation of Bi_6_O_5_(OH)_3_(NO_3_)_5_·3H_2_O nanorods, which then acted as templates for the generation of Bi_2_WO_6_. It has been observed that the newly generated Bi_2_WO_6_ has a greater BET surface area and superior charge transfer kinetics. These properties point to an increase in photocatalytic activity. Other Bi-based hollow hierarchical structures, such as BiVO_4_, have the potential to be synthesized and used as CO_2_ reduction photocatalysts. This possibility exists since these structures are hollow. It is anticipated that hierarchical Bi-based photocatalysts produced would have a broad range of applications in environmental science and energy research.

Different preparation methods have been employed for the synthesis of Bi-based photocatalysts. Each method has unique advantages and disadvantages, which are compared and contrasted in Table 1. New strategies are still required to create suitable nanomaterials by overcoming faults and enhancing the synthesis process. The hydrothermal approach is the one that is most often used to produce nanomaterials. This is because it is easy to implement and allows for a complete control over the shape and size of the nanoparticles.

**Table 1:** Various fabrication techniques, their advantages, and limitations.

No.	Technique	Advantages	Limits	Ref.

1	solid-state technique	high crystallinity, easy operation	phase transition, high temperature, big grain particle, tiny surface area	[66]
2	chemical precipitation	convenient synthesis, low cost, and low energy consumption	aggregation of particles, limited surface area, development of impurities, uncontrolled morphology	[67]
3	sol–gel technique	controllable morphology, nanoparticles, tiny and fine particles	organic residues, high costs, and treatment procedure	[68]
4	hydrolysis	different particles size, easy synthesis conditions, and simple equipment	uncontrollable morphology, solvent-dependent, poor dispersion	[69]
5	hydrothermal	simple operation, high crystallinity, a variety of morphologies, and particle size control	high pressure, different reaction parameter	[70]
6	drop casting method	convenient precipitation, easy operation	time-consuming, unpredictable thickness and homogeneity, and poor adhesion	[71]
7	dip/spin-coating	simple operation, thickness control, homogenous film	inadequate attachment, specialized equipment	[72]
8	spray pyrolysis	quick and easy operation, cost-effective, scalable technique, controlled thickness, complex composites production	high temperature, high cost, unwanted precipitates production, and high resistance	[66]
9	hydrothermal coating	easy synthesis, good conductivity, long durability, and controllable morphology	high pressure, poor yield, complex parameters, and poor dispersion	[68]
10	chemical vapor deposition	regular thickness, minimal porosity, diverse materials, and a high degree of crystallinity	extreme temperatures and specialized equipment	[66]
11	anodization technique	large-scale synthesis, large surface area, and morphological control	applied bias, complex synthesis parameters	[73]
12	electrodeposition technique	thickness control, homogenous film	specific equipment, imposed bias, and treatment procedure	[74]

### Enhancement techniques

The characteristics of a semiconductor photocatalyst alter as its size is reduced to the nanoscale. Increasing the proportion of atoms or ions exposed on the photocatalyst surface will increase the number of photocatalytically active sites [75]. Under light irradiation, the average amount of time it takes for a photogenerated carrier to diffuse from the bulk to the surface may be calculated as follows: τ = *r*^2^/π^2^*D*, where *r* is the grain radius and *D* is the carrier’s diffusion coefficient. Consequently, as the particle radius decreases, a higher number of photogenerated carriers can reach the surface, where they might participate in a photocatalytic process [76]. Bismuth is often used as a nanoscale plasmonic photocatalyst. Nanospheres, nanorods, and nanosheets can be synthesized using various techniques. Hydrothermal calcination, template synthesis, precipitation, reverse micro-emulsion, sonochemical procedures, and microwave methods are typical techniques for fabricating Bi-based nanostructures [77]. Recombination of charge carriers and insufficient photon absorption are the two most common problems related to semiconductor photocatalysts. Also, it is necessary to have a wide bandgap to yield an adequate redox ability. However, a moderate bandgap is required to perform photocatalysis using visible light. This gap is essential for improving the material’s light-harvesting capabilities [78–79]. Consequently, in an attempt to improve the photocatalytic efficiency for water purification and other environmental applications, a variety of techniques, such as defect formation, metal/non-metal doping, heterostructure formation, interface modification, and Bi-content enhancement, have been employed.

#### Defect formation

Vacancies and defects affect the electrical properties of Bi-based semiconductor photocatalysts and, hence, govern the photocatalytic efficacy. Rao et al. reported an N_2_-assisted heat treatment approach for the in situ synthesis of a series of oxygen-vacancy (OV)-rich Bi^0^/Bi-based photocatalysts [80]. A new understanding of how Bi^0^ nanoparticles and OVs are created in situ in Bi-based photocatalysts has been reported. Compared to other Bi-based photocatalysts, Bi^0^/OV–(BiO)_2_CO_3_ showed high photocatalytic performance and stability for the photooxidative elimination of NO. The ohmic interaction between OV–(BiO)_2_CO_3_ and Bi^0^ has been shown to promote the synthesis of ^•^O_2_^−^ and ^•^OH species. It was found that ^•^O_2_^−^ had a significant impact on the photocatalytic elimination of NO.

**Figure 3:**
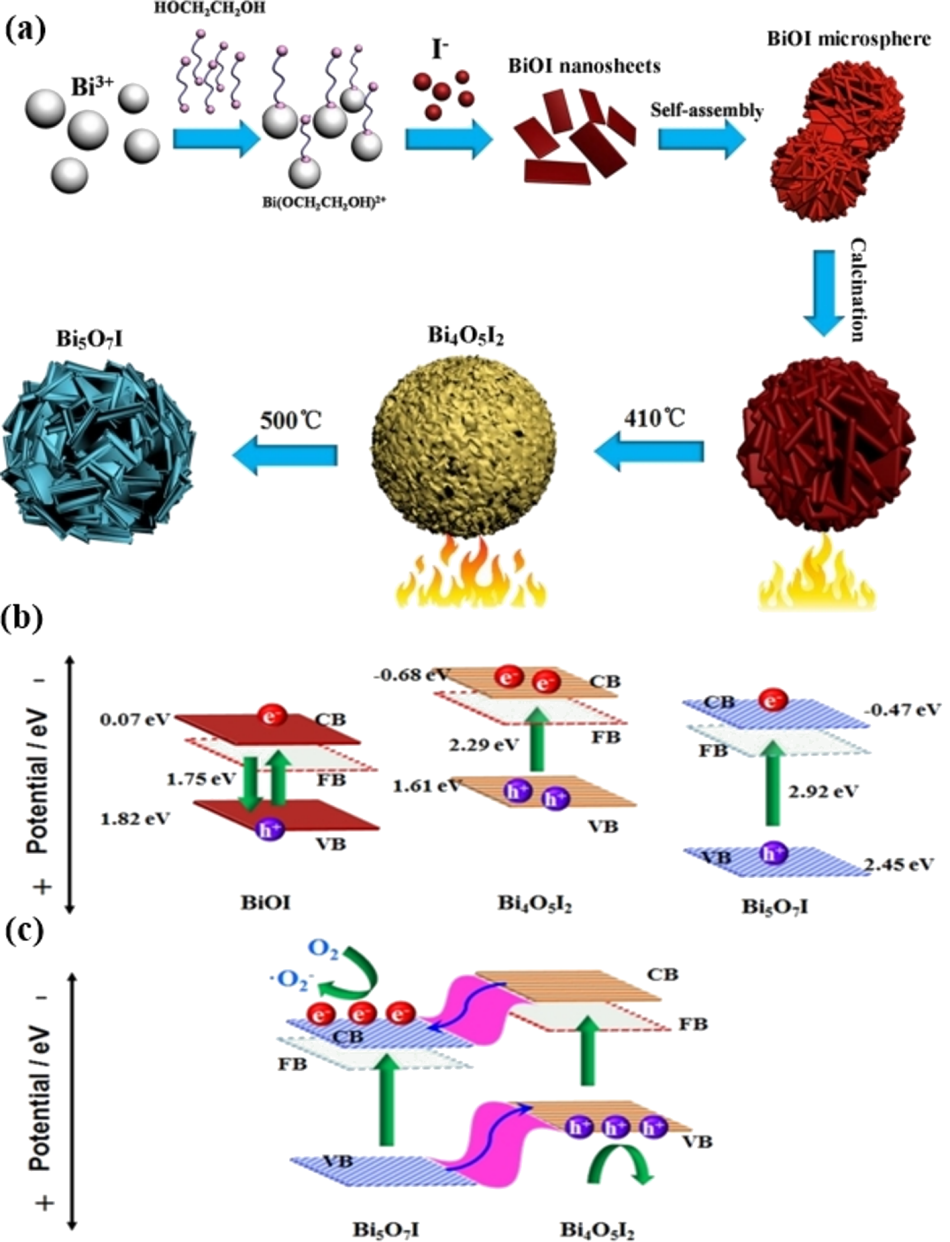
(a) The hierarchical structures of BiOI, Bi_4_O_5_I_2_, Bi_4_O_5_I_2_–Bi_5_O_7_I composite, and Bi_5_O_7_I are shown in this diagram. A schematic description of the charge segregation and transfer process for (b) the single photocatalyst of BiOI, Bi_4_O_5_I_2_, and Bi_5_O_7_I, and (c) Bi_4_O_5_I_2_–Bi_5_O_7_I phase junction. Figure 3a–c were reprinted from [81]. This article was published in Applied Catalysis B: Environmental, vol. 203, by H. Huang; K. Xiao; T. Zhang; F. Dong; Y. Zhang, “Rational design on 3D hierarchical bismuth oxyiodides via in situ self-template phase transformation and phase-junction construction for optimizing photocatalysis against diverse contaminants”, pages 879–888, Copyright Elsevier (2016). This content is not subject to CC BY 4.0.

In another approach, Huang et al. reported that BiOI microspheres served as self-sacrificing templates for in situ phase transformation and formation of phase junctions [81]. Different bismuth oxyiodides were formed as a result of this. Hierarchical BiOI, Bi_4_O_5_I_2_, Bi_4_O_5_I_2_–Bi_5_O_7_I phase-junction, and Bi_5_O_7_I may be synthesized from bismuth oxyiodides at different temperatures (Figure 3a). The photoabsorption wavelength of these bismuth oxyiodides has been tuned between 400 and 700 nm. Also, these compounds have a distinctive microstructure and a controllable band structure. (Figure 3b). The breakdown of antibiotics and pollutants such as tetracycline hydrochloride, bisphenol A (BPA), and RhB was used to measure the photocatalytic activity of the bismuth oxyiodides. The activity decreased in the sequence Bi_4_O_5_I_2_–Bi_5_O_7_I > Bi_4_O_5_I_2_ > BiOI, which is linked to charge separation efficiency and band structure. Engineered Bi vacancies in monolayered Bi_2_WO_6_ nanosheets with a thickness of 1.0 nm have recently been shown [82]. The Bi defects were shown to promote the adsorption and activation of reactant molecules, which reduced the energy barrier even more. The photocatalytic performance corroborated this. The presence of divacancies may help increase charge carrier separation by capturing photogenerated electrons close to the divacancies. In comparison, pure Bi_2_WO_6_ nanosheets had a photocatalytic performance 32 times lower for the elimination of gaseous toluene when exposed to visible light.

Recently, Zou et al. proposed a simple hydrothermal synthesis for preparing 2D BiOCl nanosheets [83]. This was accomplished by altering the pH value of the precursor solution and using of dulcitol (C_6_H_14_O_6_) as surfactant. The pH value substantially influenced the thickness of the nanosheets and the fraction of exposed (001) facets. The sample synthesized at pH 4 demonstrated outstanding visible-light photocatalytic performance regarding the degradation of RhB. This was due to the low thickness, exposed (001) facets, and an appropriate number of oxygen vacancies. This work proposed that revolutionary photoexcitation mechanisms were found on oxygen vacancies. Irradiation with visible light excites the electrons in the VB to transition into defect states. In addition, photogenerated defect states cannot readily recombine with photogenerated holes because oxygen vacancies operate as electron traps. Because of this, electrons trapped inside the oxygen vacancies have a longer lifetime than those in the CB. Therefore, electrons in defect states have the potential to react with oxygen that has been adsorbed by oxygen vacancies, which results in the production of superoxide ^•^O_2_^−^ radicals. These may subsequently be employed to drive photocatalytic processes. The absence of a metal atom would considerably affect the amount of absorbed light, the rate of charge transfer, and the number of reactive surface sites.

#### Metal/non-metal doping

Photocatalysts based on bivalent cations may be enhanced by doping them with additional elements. Self-doping and deposition and doping of metals and non-metallic elements are the most common doping methods. Metal ions modify the crystal structure of the Bi-based semiconductor photocatalysts or induce defects. Also, the photocatalytic properties may be altered by doping or deposition of metallic components [84–85]. Using a straightforward hydrothermal procedure, Hu et al. produced iron-doped Bi_2_WO_6_ nanocomposites [84].

In comparison to pristine Bi_2_WO_6_, Fe-doped Bi_2_WO_6_ exhibits superior visible-light photoabsorption, a considerably increased number of oxygen vacancies, and a noticeably improved capacity for separating photogenerated electrons and holes. As a result of the Fe doping, an impurity energy level was produced close to the VB, and an imperfection (oxygen vacancy) energy level was produced close to the CB. Both of these energy levels are in a position to potentially accept photoinduced holes and electrons, which significantly improves the electron–hole pair splitting. When Fe^3+^ substitutes W^6+^, the structure of the crystal is not compromised in any way; nevertheless, numerous additional oxygen vacancies are produced. The increased electron–hole pair separation is the driving factor behind the improved photocatalytic activity of the Fe-doped Bi_2_WO_6_ compound. When exposed to visible light, Fe-doped Bi_2_WO_6_ exhibited photocatalytic degradation rates that were 11.9 and 8.0 times higher than those of pristine Bi_2_WO_6_. This material was also found to be superior to the majority of modified Bi_2_WO_6_ photocatalysts that had been reported in the past. In addition to this, Fe-doped Bi_2_WO_6_ has a high degree of stability. The results of this study provide new information on boosting the photoactivity of Fe-doped Bi_2_WO_6_.

The SPR effect can be obtained by the deposition of metallic elements on a semiconductor surface. SPRs can potentially boost quantum yield by broadening the spectral response range of semiconductors. Fe, Au, Co, Ag, Ni, Bi, Al, and other metallic elements are often deposited and doped. For example, a nanostructure composite based on plasmonic Ag metal nanoclusters and monoclinic BiVO_4_ nanoparticles was fabricated using high-energy ball milling [86]. Ag clusters (5–10 nm) were homogeneously distributed on the flocculated BiVO_4_ particles (50–100 nm). The structure of the Ag-doped BiVO_4_ nanocomposite would promote the efficiency of the photodegradation of acid blue dyes. Huang and co-workers observed that a Bi-Bi_2_WO_6_ composite successfully degraded RhB and 4-chlorophenol under visible light [87]. They reported that the formed heterojunction yielded a two times higher RhB photodegradation and a three times higher 4-chlorophenol photodegradation than bare Bi_2_WO_6_.

Using nonmetal doping, doping energy levels may be created between CB and VB of Bi-based photocatalysts. This can increase light absorption, and charge transfer may be improved to enhance electron–hole segregation and reduce recombination. It is not uncommon to see nonmetals atoms replaced with heteroatoms, such as N (C), B (S), X (F, Cl), Br (I). Dong et al. [88] reported the fabrication of boron-doped Bi_3_O_4_Cl ultrathin nanosheets via a solvothermal technique, which were found to have enhanced solar absorption and efficient electron–hole separation. The B atoms enhance the photocatalytic performance via (1) producing mid-gap states to widen the light response region significantly up to 557 nm and (2) functioning as electron capture centers to accelerate charge carrier separation. According to ESR measurements, B-doped Bi_3_O_4_Cl can create more ^•^O_2_^−^ and ^•^OH radicals. Consequently, the B-doped sample has a 3-fold and 2.1-fold better degradation efficiency for, respectively, BPA and ciprofloxacin than pristine Bi_3_O_4_Cl. This study offers fresh perspectives on photocatalyst design and underlines the importance of electronic structure modification in catalytic activity adjustment.

**Figure 4:**
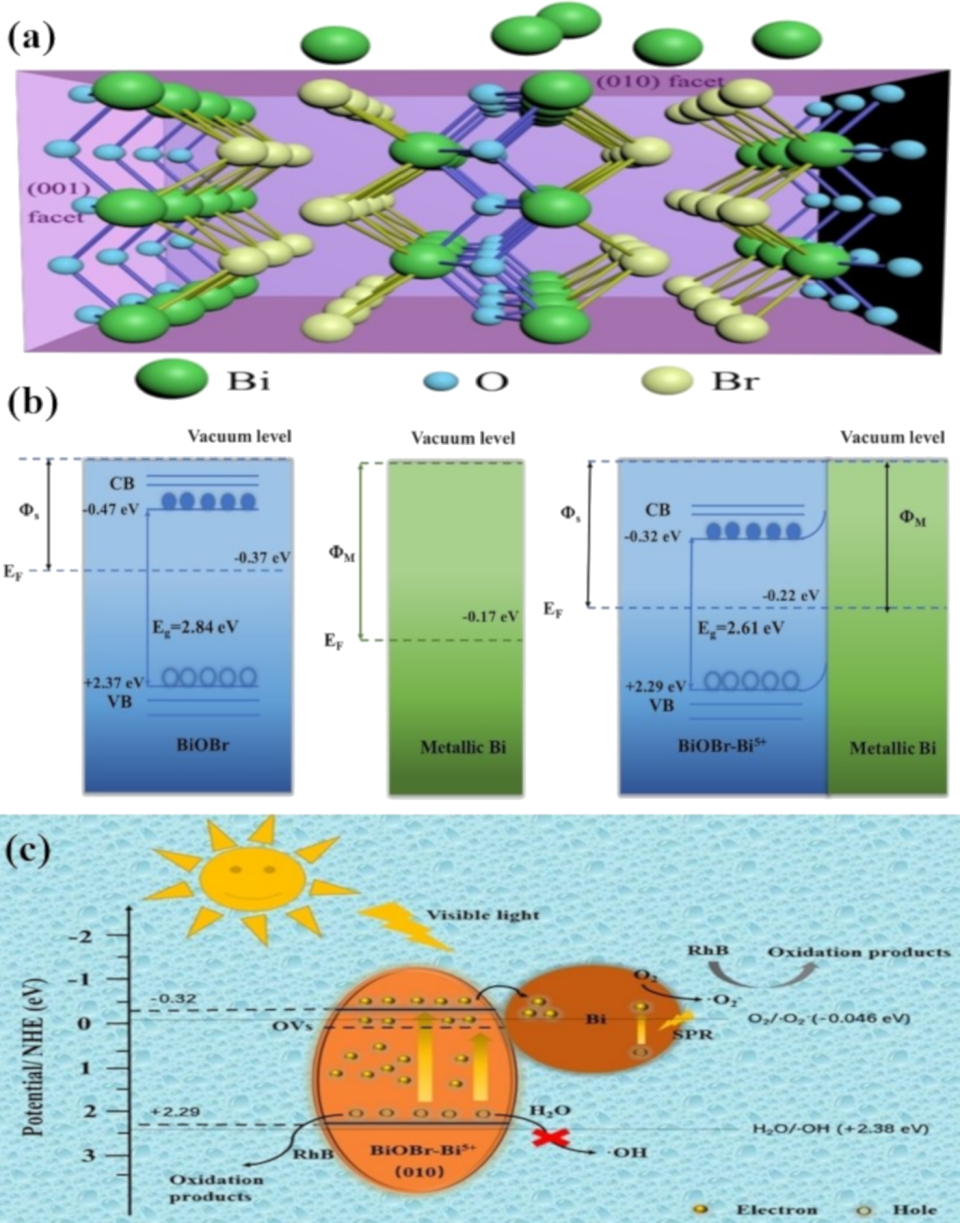
(a) Crystal structure of self-doped Bi/BiOBr–Bi^5+^, (b) band structure, and (c) RhB degradation of Bi/BiOBr–Bi^5+^ in visible light. Figure 4a–c were adapted from [90]. This article was published in Separation and Purification Technology, vol. 253, by Q. Wu; S. Chai; H. Yang; Z. Gao; R. Zhang; L. Wang; L. Kang, “Enhancing visible-light driven photocatalytic performance of BiOBr by self-doping and in situ deposition strategy: A synergistic effect between Bi^5+^ and metallic Bi”, article no. 117388, Copyright Elsevier (2020). This content is not subject to CC BY 4.0.

Self-doping is a novel approach for introducing intermediates from the synthesis process into photocatalysts to alter the energy band structure and increase photocatalytic activity [89]. A simple two-step technique was used to develop a novel compound photocatalyst of Bi/BiOBr-Bi^5+^ [90]. X-ray diffraction, field-emission transmission electron microscopy, and X-ray photoelectron spectroscopy revealed the coexistence of self-doped Bi^5+^ and in situ deposited Bi(M). Compared to Bi/BiOBr or BiOBr–Bi^5+^, the photocatalytic activity of Bi/BiOBr-Bi^5+^ regarding RhB degradation under visible light was significantly increased. OVs helped in separating photoexcited carriers, and SPRs enhanced the ability to absorb visible light. The photocatalytic activity of Bi(M) was further enhanced by the exposure of (010) facets (Figure 4a). Bi^5+^ reduced the bandgap of BiOBr, which led to an increase in the density of carriers. Examining electron transfer channels and identifying the most active species led to a credible mechanism for RhB degradation (Figure 4b,c).

It has been shown that the carrier combination centers may easily get doped at a deep level, considerably lowering photocatalytic activity. As a result, optimizing the electrical configuration by using suitable dopants and concentrations will increase photocatalytic activity. Photogenerated electron–hole separation efficiency and light absorption ability are critical functions that can be improved by appropriate doping.

#### Heterojunction formation

**Figure 5:**
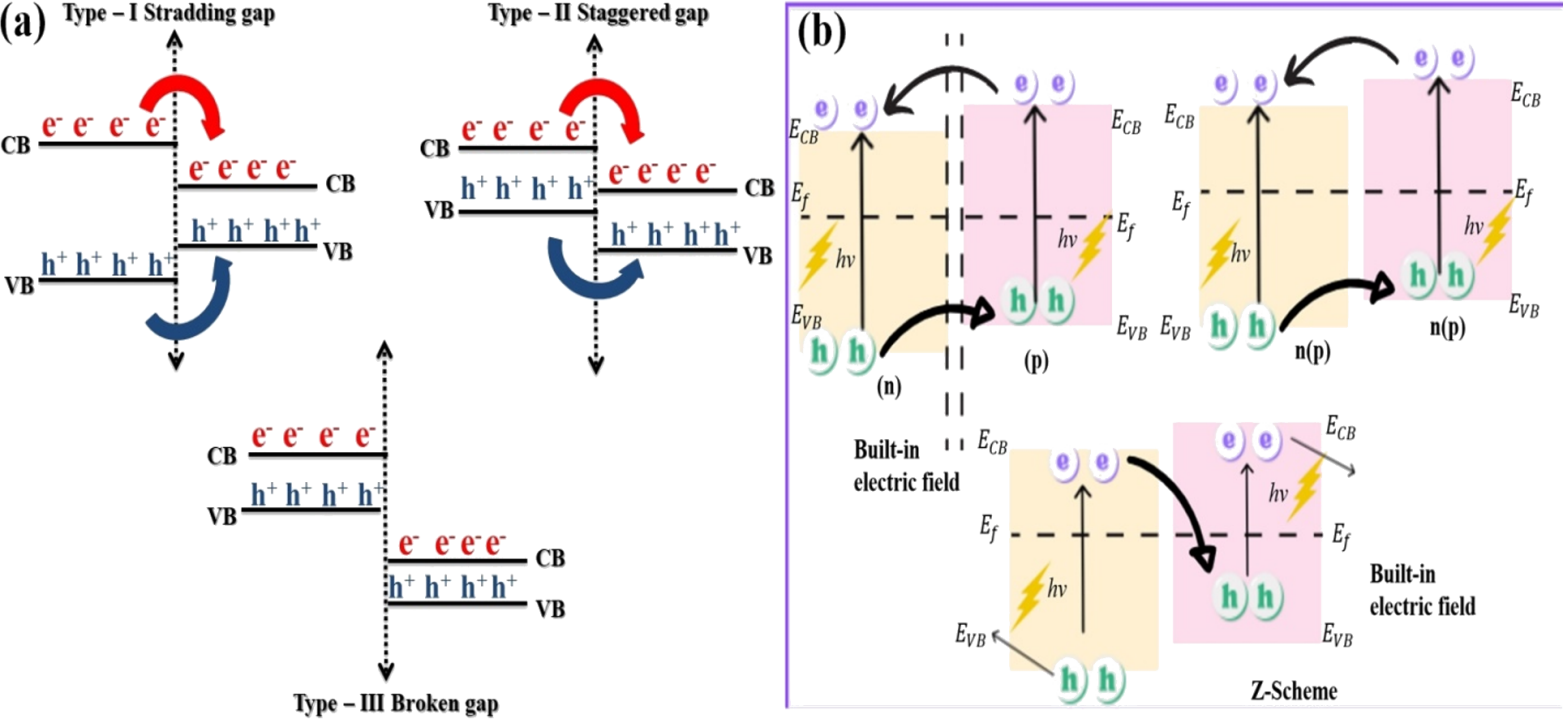
(a) Band diagrams representing three different semiconductor heterojunctions, (b) band diagrams for p–n, n–n (p–p), and Z-scheme-based heterostructures.

In general, for a photocatalyst to function appropriately, it is necessary to use a semiconductor with the following characteristics: a bandgap suitable for light harvesting, effective charge carrier separation capabilities, and suitable VB and CB edge potentials [91–92]. It is challenging to meet these requirements with only one single Bi-based photocatalyst. Constructing semi-conductor heterojunctions may be an effective technique for addressing the difficulties of individual Bi-based photocatalysts. This may be due to the changeable band structure and effective photoinduced electron–hole separation, which bestows them with higher capabilities [93]. It is possible to improve absorption of visible light, charge carrier segregation, and charge transport effectiveness by combining heterojunction and nanomaterials in photocatalysts constructed with care. As a result, this technique offers much potential for photocatalysis applications [94]. The band arrangement shown in Figure 5a may be used to classify the heterojunctions between semiconductors as either a straddling gap (type I), an uneven gap (type II), or a broken gap (type III). Type-II heterojunctions have garnered the most interest because of the improved photogenerated electron–hole separation they offer. They include n–n heterojunctions, p–n heterojunctions, p–p heterojunctions, and Z-scheme-based heterojunctions (Figure 5b). By using heterojunctions, it is possible to exert control over the electronic components of the photocatalyst to increase light absorption and photoinduced separation and migration [95–96]. Photoinduced holes in n-type semiconductors are transported to p-type semiconductors by an electric field at the interface, whereas photoinduced electrons from p-type semiconductors are transported to n-type semiconductors (Figure 5b).

Using simple and cost-effective experimental conditions, Sang et al. reported the fabrication of nanoflower-like Bi_2_O_3_/Bi_2_S_3_ heterojunctions via a one-step hydrothermal technique [97]. In the photocatalytic elimination of RhB and Cr(VI) under visible-light irradiation, the photocatalytic activity of this Bi_2_O_3_/Bi_2_S_3_ heterojunctions outperforms that of pristine Bi_2_O_3_ and Bi_2_S_3_. They found that photoinduced holes were the main oxidative species for eliminating RhB, whereas photogenerated electrons were responsible for the photoreduction of Cr(VI). Typically, the Fermi energy level of n-type Bi_2_O_3_ is quite close to the VB, while the Fermi energy level of p-type Bi_2_S_3_ is somewhat close to the CB. In a heterojunction from n-type Bi_2_O_3_ and p-type Bi_2_S_3_, electrons flow from n-type Bi_2_O_3_ to p-type Bi_2_S_3_, and holes flow from p-type Bi_2_S_3_ with a low Fermi level to n-type Bi_2_O_3_ with a high Fermi level. As a consequence of this, negative charges build up in Bi_2_S_3_ near the interface, which leads to an electric field. Also, the Fermi level of Bi_2_O_3_ goes down, while the level of Bi_2_S_3_ goes up. Along the Fermi level, the energy bands of both Bi_2_O_3_ and Bi_2_S_3_ are moving simultaneously in a downward and an upward direction. An equilibrium state, in which the Fermi levels of Bi_2_O_3_ and Bi_2_S_3_ equilibrate at the p–n junction, was also proposed. Bi_2_S_3_, which has a smaller bandgap than Bi_2_O_3_, is excited when it is exposed to visible light, while Bi_2_O_3_ is not. The photogenerated holes stay in the p-type Bi_2_S_3_ VB, while the excitons in the p-type Bi_2_S_3_ CB migrate to the n-type Bi_2_O_3_ CB. In the Bi_2_O_3_/Bi_2_S_3_ photocatalytic system, electrons and holes participate directly in the redox process. In another reported work, a BiOI/Bi_2_O_2_CO_3_/graphene ternary composite was fabricated via a facile and economic hydrothermal technique [98]. In order to assess the newly constructed semiconductor heterojunction, tetracycline and RhB were degraded using visible light. Better photocatalytic activity can be achieved with BiOI/Bi_2_O_2_CO_3_ photocatalysts instead of just using BiOI alone. This is because in BiOI/Bi_2_O_2_CO_3_ photocatalysts, an electric field emerges at the p–n heterojunction, which in turn helps to foster the movement of photogenerated carriers. Furthermore, the high photocatalytic activity of the BiOI/Bi_2_O_2_CO_3_/RGO composite can be attributed to the fact that the positively charged BiOI/Bi_2_O_2_CO_3_ was electrostatically paired with the negatively charged graphite oxide (GO) to form interlayer contacts. This caused the photocatalytic reaction sites to boost, the light response to broaden, and the separation of photoinduced charge to improve. Lv et al. [99] fabricated a p–n heterojunction-based novel CuS/Bi_2_WO_6_ semiconductor photocatalyst with 2D interfacial connections of CuS over the surface of Bi_2_WO_6_. The hydrothermal method was used, and it was discovered that the produced CuS/Bi_2_WO_6_ semiconductor photocatalyst had increased photocatalytic performance for the breakdown of glyphosate when exposed to visible light. Enhanced photocatalytic activity, excellent recyclability, high stability of CuS/Bi_2_WO_6_ photocatalysts may be primarily due to the presence of an electrical potential at the interface, which is responsible for both the enhanced visible light absorption and the efficient segregation of photoinduced charges. This study introduced a unique 2D interfacial coupling for the production and design of efficient photocatalysts. It also demonstrated that CuS may be used as an effective semiconductor in heterostructures, which is a concept that may be extended to other functional nanomaterials based on bismuth.

**Figure 6:**
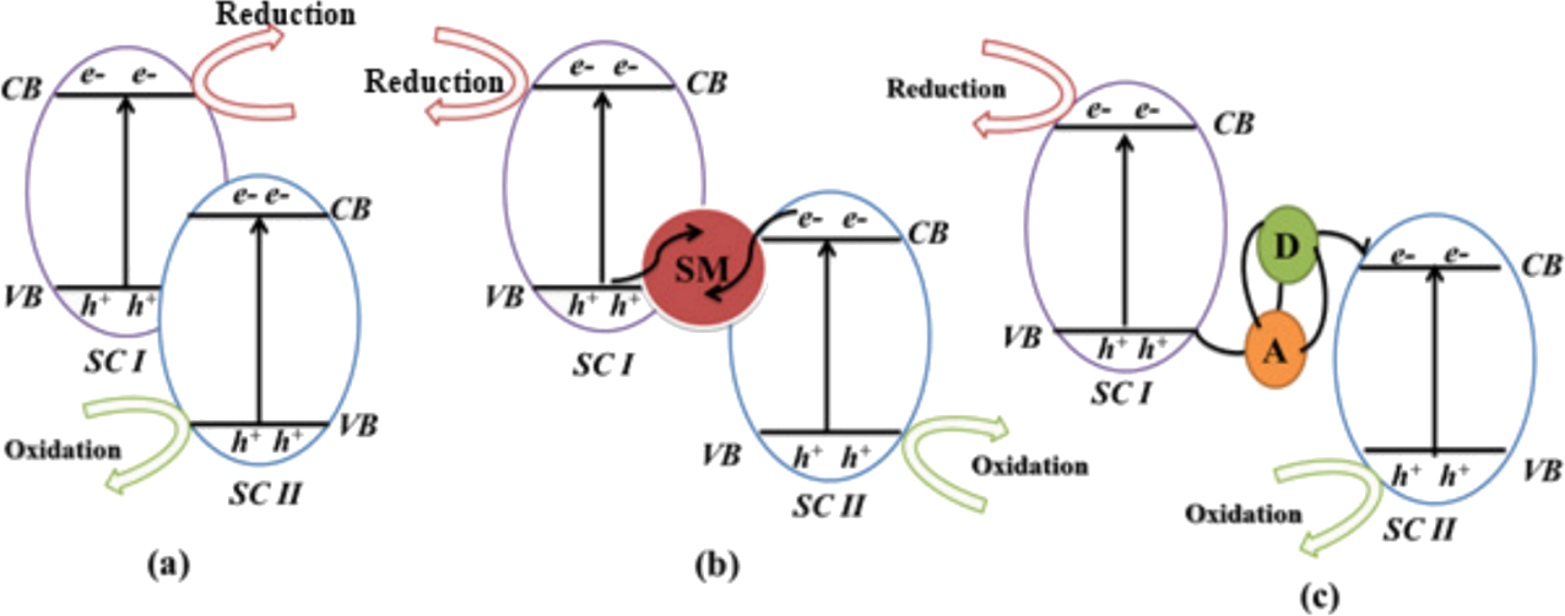
Different charge transfer Z-scheme types: (a) Mediator-free or direct, (b) solid mediator, and (c) redox pair mediator. Abbreviation lists: semiconductor I (SC I), semiconductor II (SC II), valence band (VB), and conduction band (CB).

The most important heterojunctions are known as Z-scheme heterojunctions and have a band energy structure comparable to type-II heterojunctions. However, there are many alternative pathways open for charge carrier movement (Figure 6), including mediator-free or direct (Figure 6a), solid mediator (Figure 6b), and redox pair mediator (Figure 6c) [43,100]. A “mediator” is often employed to offer an intermediary conduit for electrons to flow from the CB of semiconductor II (SC II) to the VB of semiconductor I (SC I), making the charge transfer easier [38]. A solid substance or a redox couple in solution may serve as a mediator in the Z-scheme. This dual absorber system has the potential to get excited, which would result in the generation of photogenerated holes and electrons in CB and VB, respectively. Photogenerated electrons in the CB of SC II may migrate to the VB of SC I, where they may merge with photogenerated holes. This design is favorable for the electrons in the CB of SC I and the holes in the VB of SC II to keep their optimum and original redox potentials (Figure 6a). This method of electron transmission resembles the letter Z. As a result, the structure of this heterojunction is referred to as the direct Z-scheme [43,101]. Fu et al. [102] demonstrated the synthesis of a new direct Z-scheme photocatalyst made of ultrathin Bi_2_O_3_ and Bi_2_MoO_6_ microspheres. For the effective production of Bi_2_O_3_/Bi_2_MoO_6_ nanocomposites, researchers adopted a simple in situ alkali treatment of Bi_2_MoO_6_ followed by calcination. As a substrate for the production of Bi_2_O_3_ sheets, Bi_2_MoO_6_ microspheres were used. The 2D morphological properties of the Bi_2_O_3_ sheets resulted in enhanced charge carrier transfer. The relative mass ratio of Bi_2_MoO_6_ and Bi_2_O_3_ may be fine-tuned by adjusting the alkali dose (i.e., NaOH or KOH). Using phenol degradation and hydrogen generation as a test, the 1.5% Bi_2_O_3_/Bi_2_MoO_6_ sample was shown to be the most photocatalytically active. An easy hierarchical Z-scheme system with a ZnIn_2_S_4_/BiVO_4_ heterojunction has been proposed by Hu and co-workers [103]. This system can precisely regulate redox centers at the ZnIn_2_S_4_/BiVO_4_ heterojunction by expediting the detachment and mobility of photoinduced charges. This, in turn, increases the ability of holes and electrons to undergo oxidation and reduction, respectively. As a consequence of this, the ZnIn_2_S_4_/BiVO_4_ heterojunction has unusual photocatalytic activity. It has an H_2_ evolution rate much higher than pure ZnIn_2_S_4_, with a value of 5.944 mol·g^−1^·h^−1^. This value is over five times higher. In addition, this heterojunction has excellent stability and the capacity to be recycled, making it a promising photocatalyst for the formation of H_2_. Ternary composite heterojunction photocatalysts have been also reported. The Fenelon group successfully fabricated SnO_2_ and Bi_2_S_3_–Bi_25_ composites via a facile hydrothermal technique followed by thermal breakdown [27]. The photocatalytic performance of the fabricated ternary composite photocatalyst was 2.75 times higher than that of pristine Bi_2_S_3_ for the photodegradation of RhB under visible light. Their research also revealed that 15% SnO_2_ precursor solution was the most effective concentration for achieving a photocatalytic degradation efficiency of 80% after 180 min of exposure to visible light. Photogenerated holes were found to be responsible for the oxidation and breakdown of the pollutant during the photocatalytic reaction.

One of the most popular ways to create an effective heterojunction structure is by combining two semiconductors with matching band alignment. When it comes to understanding the mechanisms behind the enhanced photocatalytic performance of heterojunction photocatalysts, researchers have recently proposed an S-scheme heterojunction [45]. Separating photoinduced electrons and holes with an S-scheme heterojunction efficiently preserves the promising redox properties of semiconductors. It is common for electrons to flow from the CB of one semiconductor to the VB of the other in an S-scheme heterojunction photocatalyst due to an IEF that typically exists at the interface of the two semiconductors [104]. Lately, Xu et al. reported the fabrication of a MoS_2_/BiVO_4_ heterojunction via solvothermal and electrospinning techniques [105]. Without any additional agent, the fabricated heterojunction completely degraded a RhB dye solution within 20 min. BiVO_4_ nanorods photogenerated hydroxyl radicals rather than super oxides because of the more positive oxidation potential of BiVO_4_ (2.31 V). MoS_2_ sheets favored the photogeneration of superoxide radicals because of the more negative CB position (−1.39 V). This finding demonstrated the spatial distribution of oxidation sites (BiVO_4_) and reduction sites (MoS_2_) via an S-scheme charge transfer path and significantly aided in the inactivation of bacteria under illumination. In another work, a simple hydrothermal technique was applied by Liu et al. to modify BiVO_4_ heterojunctions with carbon quantum dots [106]. Exceptional photocatalytic performance for the degradation of RhB dye under visible light was exhibited by CNQDs-ms/tz-BiVO_4_ and NCQDs-ms/tz-BiVO_4_ composites. The reason for their increased photocatalytic efficiency was the formation of heterojunctions together with the loading of quantum dots, which enhanced the light-harvesting efficiency and promoted the separation and migration of photogenerated carriers. The fabricated composites followed the S-scheme charge transfer mechanism, efficiently contributing to enhanced photocatalytic performance. Another research group reported that photoreduction and hydrothermal techniques were used to successfully synthesize a new 2D/2D Bi_2_MoO_6_/g-C_3_N_4_ S-scheme composite including Au as a co-catalyzer [107]. Bi_2_MoO_6_/g-C_3_N_4_/Au had a photocatalytic activity in RhB degradation that was 9.7 times and 13.1 times higher than that of Bi_2_MoO_6_ and g-C_3_N_4_, respectively. In the Bi_2_MoO_6_/g-C_3_N_4_/Au system, the higher photocatalytic activity can be attributed to the abundance of active sites and the enhanced separation efficiency of photogenerated carriers. The potential role of Au nanoparticles in the S-scheme heterostructure is noteworthy. They serve as a co-catalyst for improving electron separation and transmission due to the photogenerated potential.

By forming heterojunctions, the visible-light absorption as well as the carrier separation efficiency of Bi-based nanophotocatalysts can be improved, which in turn increases the photocatalytic activity. The zonal organization of several crystalline surfaces made from the same material may significantly increase charge separation. It can be used to create classic heterojunctions in addition to surface heterojunctions, which can be created using the same method. Surface heterojunctions have recently emerged as a novel concept that has garnered much interest. As a direct consequence of this, it is possible that in the not-too-distant future, it will be possible to construct and analyze further Bi-based surface heterojunction photocatalysts. Table 2 below provides an in-depth explanation of several different strategies that may be used to improve Bi-based nanocomposites.

### Environmental applications

The use of Bi-based photocatalysts in power production and environmental remediation is widespread. Sewage treatment, environmental monitoring, disinfection, and sterilization are all areas where the photocatalytic breakdown of contaminants is used. Primary energy uses included photocatalytic hydrogen production from carbon dioxide, conversion of carbon dioxide to specific molecular organic matter, and nitrogen fixation [1,108–109]. Photocatalysts with Bi-based photocatalysts are discussed in this section in more depth.

#### Photocatalytic hydrogen generation

At the moment, humanity’s existence greatly depends on the consumption finite fossil fuels. Thus, finding long-term renewable energy sources is critical. Hydrogen is produced primarily by the electrolysis of water, in parts using solar energy, and the reformation of fossil fuels [110]. The conversion of water into hydrogen by using solar energy is considered the best way to produce hydrogen [111]. Photocatalysts still face the following issues regarding efficient water splitting. First, the quantum efficiency deteriorates as one moves into the visible region. Second, photocatalysts such as sulfides and nitrogen oxides in the visible region have poor stability and are frequently inactivated by photocorrosion. Third, the removal of O_2_ from the surface of a semiconductor photocatalyst is complex, which is why a h^+^-capturing substance is frequently added to the system to promote the production of H_2_ [112]. Pt, Pd, Au, and other precious metals, as well as photocatalysts containing Cd, Pb, and other elements (such as CdS), are some of the costliest and environmentally hazardous high-performance photocatalysts on the market. CdS is a more ecologically friendly alternative to other photocatalysts [113]. To improve the efficiency of photocatalytic water breakdown from the perspective of catalytic reaction kinetics, one method that is both practical and effective is to design photocatalysts in such a way as to change the processes involved in photocatalytic reaction kinetics.

**Figure 7:**
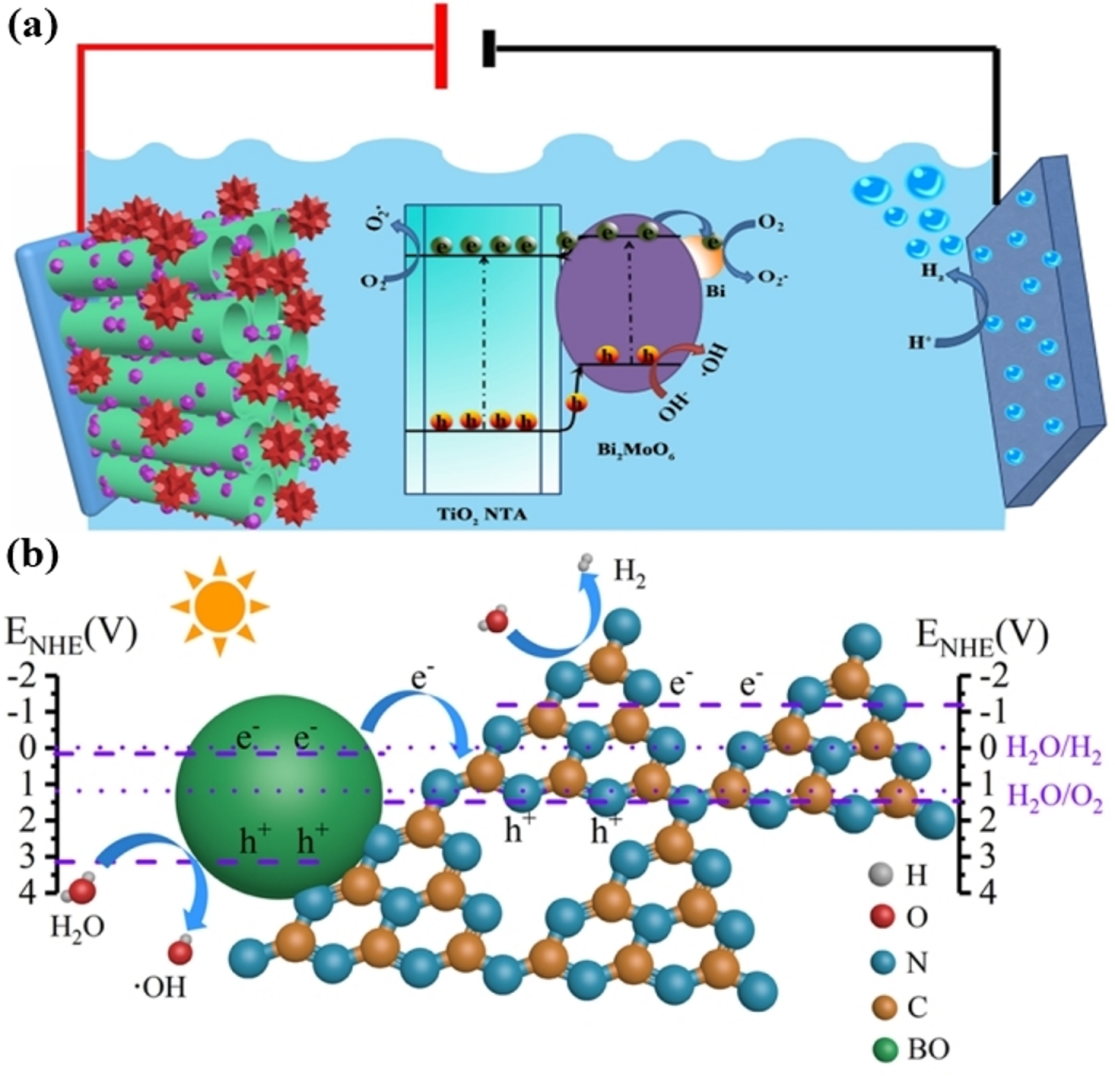
Graphical representation of the proposed charge transfer mechanism of (a) the Bi/Bi_2_MoO_6_/TiO_2_ nanocomposite and (b) the (BiO)_2_CO_3_/g-C_3_N_4_ heterojunction. Figure 7a was reprinted from [114]. This article was published in Separation and Purification Technology, vol. 250, by D. Cao; Q. Wang; Y. Wu; S. Zhu; Y. Jia; R. Wang, “Solvothermal synthesis and enhanced photocatalytic hydrogen production of Bi/Bi_2_MoO_6_ co-sensitized TiO_2_ nanotube arrays”, article no. 117132, Copyright Elsevier (2020). This content is not subject to CC BY 4.0. Figure 7b was reprinted with permission from [115], Copyright 2019 American Chemical Society. This content is not subject to CC BY 4.0.

For example, Cao et al. reported the fabrication of a bismuth-based Bi/Bi_2_MoO_6_/TiO_2_ nanocomposite photocatalytic material using a facile one-step solvothermal technique [114]. To reduce metal Bi on the surface of Bi_2_MoO_6_, they employed glucose as a reducing agent. To test the photocatalytic activity, they exposed the as-prepared materials to simulated solar irradiation and measured the degradation efficiency of RhB (7.321%), MB (92.98%), and Cr(VI) (70.54%). The highest hydrogen generation rate was 173.41 mol·h^−1^·cm^−2^, and after four cycles with the same parameters, there was no noticeable decrease in the hydrogen production of the Bi/Bi_2_MoO_6_/TiO_2_ system. The development of Bi_2_MoO_6_/TiO_2_ heterojunctions, the SPR effect of Bi, and synergistic effect may be responsible for the enhanced photocatalytic activity and hydrogen generation rate (Figure 7a). Developing semiconductor photocatalysts that are both low-cost and highly efficient is essential for the practical conversion of solar energy into fuel. A Ag–C_3_N_4_-modified (BiO)_2_CO_3_ semiconductor photocatalyst was synthesized using an in situ thermal approach in [115]. The activity of the (BiO)_2_CO_3_/g-C_3_N_4_ heterojunction reached 965 μmol·h^−1^·cm^−2^ after 5 h. This is almost three times the activity of pristine g-C_3_N_4_ or any other modified g-C_3_N_4_ nanomaterial (337 μmol·h^−1^·cm^−2^). An increase in photocatalytic activity has been found due to the usage of a direct Z-scheme system (Figure 7b). Theoretical simulations showed that charge carriers were redistributed at the junction between (BiO)_2_CO_3_ and g-C_3_N_4_.

#### Photocatalytic elimination of water pollutants

A wide variety of contaminants in water, such as heavy metal ions, pharmaceuticals, and pesticides, have been used as models substances for photocatalytic degradation. Reactive oxygen species (ROSs) such as ^•^O_2_^–^ and ^•^OH are critical to the photocatalytic removal of environmental pollutants.

Malathi et al. reported the photocatalytic and photoelectrochemical performance of BiFeWO_6_/BiOI nanohybrids, which were fabricated by wet impregnation, under visible-light irradiation [116]. Within 90 min, the composite degraded 92% of RhB. Similarly, Priya et al. used a simple wet-chemical method to make BiFeWO_6_/WO_3_ nanocomposites [117]. A 250 W tungsten halogen lamp was used to carry out the photoexcitation of the catalysts for RhB degradation. After a period of 60 min, the composite yielded a 83% breakdown of RhB. The multiferroic single-phase material known as nanoscale zero-valent iron-doped bismuth ferrite (NZVI@BiFeO_3_) displays concurrent ferroelectric, ferromagnetic, and ferroelastic properties. BiFeO_3_ has the ability to be utilized in heterogeneous photocatalysis when exposed to light from the sun. The NZVI@BiFeO_3_/g-C_3_N_4_ nanocomposite was produced using a straightforward hydrothermal process [118]. The composite was employed for the photodegradation of RhB. The generated composite could degrade 97% of the RhB dye when exposed to solar light. Compared to pure BiFeO_3_, the NZVI@BiFeO_3_/g-C_3_N_4_ composite has a better optical responsiveness, which contributes to the improved photocatalysis of the material. According to the theorized dye degradation mechanism, the composite provides efficient reactive species trapping sites. Because of this, the NZVI@BiFeO_3_/g-C_3_N_4_ nanocomposite has the potential to be utilized to oxidize a wide range of organic and inorganic contaminants that are found in wastewater.

Recently, Chava et al. proposed the solvothermal method for preparing 1D/0D CdS/Bi heterostructures [119]. To evaluate their potential photocatalytic performance toward photodegradation of the organic pollutants, tetracycline was used as model substance. The 1D/0D CdS/Bi heterostructures exhibited excellent catalytic activity, compared to pure CdS. It was expected that 1D/0D CdS/Bi could act as an effective photocatalyst for the treatment of other organic pollutants in wastewater, such as organic dyes and pharmaceuticals. The enhanced photocatalytic degradation efficiency may be due to doped Bi^3+^ species, the SPR effect in the metallic Bi quantum dots, and increased photoinduced charge carrier separation. In another study, a visible-light-sensitive heterostructure made of InVO_4_/Bi_2_WO_6_ nanoflakes was synthesized using an in situ hydrothermal process [120]. The photocatalytic degradation of tetracycline was successfully investigated. Notably, the InVO_4_/Bi_2_WO_6_ semiconductor photocatalyst manufactured with 5.0 mg of Bi_2_WO_6_ exhibits the highest tetracycline degradation rate, that is, 97.42% in 72 min. Following the findings of quenching studies, hydroxyl radicals and holes prevail throughout the photocatalytic process. In addition, the improved nanocomposite does not lose its stability after being exposed to light for four cycles, highlighting the excellent reusability and photostability of the photocatalyst.

#### Photoreduction of carbon dioxide

The CO_2_ content in the atmosphere has risen from 280 to 408 ppm since the late 1800s. Officials and scholars are now concentrating their efforts on devising strategies that would significantly reduce atmospheric CO_2_ concentrations. The photochemical reduction of carbon dioxide to convert it to hydrocarbon fuels is one of the most promising options that have been found. Energy may be obtained from sustainable solar energy, which can be used directly or indirectly in this process, resulting in carbon recycling that lives up to its name since it can be conducted outside at ambient temperature and atmospheric pressure [121–122]. Therefore, using photocatalysis to remove excess CO_2_ from the atmosphere is of the utmost significance.

However, the photocatalytic reduction of CO_2_ is difficult for several reasons. First, breaking the bonds in CO_2_ requires significant energy. Second, reducing CO_2_ to methanol or methane requires processes involving the transfer of, respectively, six or eight electrons. These processes are far more intricate than the process transferring four electrons during the breakdown of water. Third, transforming carbon dioxide into methanol and other fuels requires complex, multi-step intermediate processes, such as proton transfer and hydroxylation. Fourth, in a system that exists in a liquid phase, the reduction of CO_2_ is typically followed by a competitive reaction (proton reduction) [123]. Furthermore, CO_2_ reduction produces a range of by-products, making catalyst selectivity crucial.

Among the candidates for CO_2_ photoreduction, bismuth-based photocatalysts have received enormous attention [124]. They offer many advantages, such as excellent bandgap features and a unique electronic structure. The vacancy defects in Bi-based photocatalysts could facilitate the adsorption and activation of CO_2_, resulting in further enhancing the efficiency of CO_2_ reduction. Moradi et al. combined sol–gel and photodeposition processes to fabricate Pt@Bi-TiO_2_ photocatalysts for converting CO_2_ [125]. As expected, methane production was noticeably improved over the Pt@Bi-TiO_2_ photocatalyst. Its methane yield was about 6.2 times greater than that of pure TiO_2_. The enhanced activity might be attributed to the efficient charge separation facilitated via Pt nanoparticles, and the increase of CO_2_ adsorption on phases containing Bi. Lui et al. [126] reported that a simple in situ one-step combustion approach was used to prepare Bi_2_Al_4_O_9_/β-Bi_2_O_3_ heterojunction. Urea was employed as the fuel for the reaction, while bismuth nitrate pentahydrate was used as a source of Bi_2_Al_4_O_9_ and β-Bi_2_O_3_. A transfer pathway of the photogenerated charges was suggested. Enhancement in CO yield was ascribed to oxygen vacancies, which improved the adsorption activation of CO_2_, and the photogenerated charge carriers effectively separated in the heterostructure. The CO_2_ photoreduction performance of the heterojunction of 0.14 Bi_2_Al_4_O_9_/β-Bi_2_O_3_was determined to be the highest. In contrast to the 1.5 μmol·g^−1^ CO yield on β-Bi_2_O_3_, the sample of 0.14 Bi_2_Al_4_O_9_/β-Bi_2_O_3_ gave 13.2 μmol·g^−1^ CO. Bi integration into TiO_2_ and subsequent loading of Pt on its surface led to a significant rise in methane generation. Methane yields were about 6.2 times higher with modified TiO_2_, which included 3 wt % Bi and 1.5 wt % Pt, compared to pure TiO_2_. Pt nanoparticles facilitated charge separation and boosted CO_2_ adsorption on phases containing Bi, which increased CO_2_ conversion activity.

#### Photocatalytic nitrogen fixation

Regarding the synthesis of NH_3_, photocatalytic N_2_ fixation (2N_2_ + 6H_2_O + light → 4NH_3_ + 3O_2_) is a promising concept. To further promote N_2_ photofixation, many studies have focused to develop effective photocatalysts.

Rong et al. proposed a Bi_2_Te_3_/BiOCl heterostructure as an effective candidate for N_2_ photofixation [127]. An NH_3_ release rate of 315.9 μmol·L^−1^·h^−1^ under UV-light irradiation was achieved. The improvement might be attributed to the effective prevention of hole–electron recombination. Bi_2_Te_3_ is a highly active semiconductor with a bandgap of 0.15 eV; however, its reduction potential of 0.57 eV is less than that of N_2_/NH_3_ (0.092 eV). As its CB potential (1.1 eV) is more negative than that of N_2_/NH_3_ (0.092 eV), BiOCl may photogenerate electrons and holes under UV light. So, the electrons photogenerated from BiOCl were crucial to the N_2_ photofixation. The photocatalytic activity of BiOCl is severely hampered by the recombination of photogenerated holes and charges. The photogenerated charges from the excitation of Bi_2_Te_3_ effectively suppress the recombination of photogenerated holes and electrons of BiOCl, which extends the lifetime of electrons in the CB of BiOCl. Without the presence of light or photocatalysts, no NH_3_ was measured. Fei et al. utilized graphene quantum dots and Bi_2_WO_6_ to construct a heterostructure for photocatalytic nitrogen fixation [128]. The photocatalytic performance was, respectively, 33.8 and 8.88 times better than that of pristine graphene quantum dots and Bi_2_WO_6_. Zhou et al. used an in situ bismuth reduction technique on Bi_2_WO_6_ [129]. Metallic Bi was used as a lattice junction to build Bi_2_WO_6_, which was highly oriented on the lattice structure. The directed interface and transfer channels made separating photogenerated carriers possible, leading to successful nitrogen fixation. In another work, Zhang et al. coated Bi_2_WO_6_ with cyclized polyacrylonitrile (c-PAN), which yielded active sites for N_2_ absorption and activation because the pyridinic N atom in c-PAN shifted into an unsaturated state to interact with small molecules and to transfer electrons to the small molecules [130].

#### Photocatalytic microbial disinfection

Heat and UV light are two methods to kill bacteria and viruses. Photocatalytic technology has also been used to disinfect and sterilize air, with positive results compared to traditional methods. Compared to TiO_2_ and ZnO, bismuth-based photocatalysts have a small bandgap and better visible light absorption [131]. Wang et al. reported a hydrothermally synthesized monoclinic dibismuth tetraoxide (m-Bi_2_O_4_) with a small bandgap of 2.0 eV and mixed valence states (Bi^3+^ and Bi^5+^) [132]. After 120 min of visible light irradiation, the m-Bi_2_O_4_ nanorods inactivated *E. coli* with a substantially better photocatalytic efficiency and photostability than CdS and Bi_2_O_3_. The primary ROS responsible for photocatalytic disinfection was found to be ^•^OH. This research also suggested that bismuth-based nanomaterials might be effective, stable, and long-lasting semiconductor photocatalysts for water disinfection under visible light. In another study, Liang et al. reported the fabrication of AgI/AgBr/BiOBr_0.75_I_0.25_ nanocomposites via solvothermal technique [133]. The fabricated semiconductor photocatalyst was used to deactivate *Escherichia coli* under visible light. A concentration of 80 mg/L AgI/AgBr/BiOBr_0.75_I_0.25_ was able to totally inactivate 3 × 10^7^ CFU·mL^−1^ *E. coli* cells in 30 min. Furthermore, the bactericidal processes were thoroughly explored. The bactericidal activity of Ag^+^ ions generated from the nanocomposite was negligible, whereas active species such as h^+^, e^−^, and ^•^O_2_^−^ played critical roles in the disinfection. Direct interaction between bacterial cells and nanoparticles was discovered to be necessary for both the production of ^•^O_2_^−^ and disinfection processes. *E. coli* cells were inactivated by disrupting the cell membrane and releasing cytoplasm. Furthermore, even after four repeated cycles, AgI/AgBr/BiOBr_0.75_I_0.25_ showed excellent antibacterial activity against *E. coli*. A novel Z-scheme AgI/BiVO_4_ heterojunction was synthesized via the chemical deposition–precipitation technique [134]. *E. coli* disinfection and the decomposition of oxytetracycline hydrochloride (OTC-HCl) were used to measure the photocatalytic activity under visible-light irradiation. The Z-scheme heterojunction took 50 min to kill bacteria and degraded OTC-HCl by 80% via photocatalysis, exhibiting high photocatalytic performance and photostability. h^+^ and ^•^O_2_^−^ were shown to be the major reactive species during photoinactivation, with K^+^ permeability playing an important role in cell membrane collapse and bacterial deactivation. It was shown that the AgI/BiVO_4_ photocatalyst is an effective nanomaterial for wastewater treatment, specifically with extremely high concentrations of pathogenic microorganisms and antibiotics. Table 2 lists a Bi-based nanomaterials that have been studied for environmental applications.

**Table 2:** Different roles of Bi-based semiconductor photocatalysts in various environmental remediation applications.

No	Photocatalyst	Enhancement strategy	Environmental Application	Role of photocatalyst	Ref.

1	BiOBr	facet-dependent	bacterial disinfection	enhanced photocatalytic generation of radical	[135]
2	Bi_2_O_4_/BiOBr nanosheets	facet-dependent	MO dye degradation, microbial disinfection	enhanced light absorption, efficient photoinduced e^−^/h^+^ pair separation, boosted surface-adsorbed ability	[136]
3	BiO_2−_*_x_*	Z-scheme heterojunction	O_2_ evolution	increased IEF	[137]
4	Bi_2_MoO_6_/g-C_3_N_4_	heterojunction formation	H_2_ evolution, microbial disinfection	enhanced reduction capabilities	[138]
5	BiOI/BiOBr	heterojunction structure	bacteriostatic activity	advance separation of the photoinduced holes	[139]
6	AgI/Bi_2_MoO_6_	Z-scheme	water disinfection	strong redox potential and enhanced separation of photogenerated charge carriers	[140]
7	MoS_2_/Bi_2_WO_6_	p–n junction formation	water detoxification	generated electric field, efficient separation of photogenerated charges	[141]
8	BiOCl	surface defects	CO_2_ photoreduction	increased surface adsorption	[142]
9	Bi_4_O_5_Br_2_	Bi-rich strategy	CO_2_ photoreduction	efficiently improved surface properties	[143]
10	Bi_2_WO_6_/CuBi_2_O_4_	Z-scheme formation	tetracycline dye degradation	increased surface oxygen vacancy	[144]
11	BiPO_4_/BiOCl_0.9_I_0.1_	Z-scheme	phenol and RhB dye degradation	boosted redox ability increased charge transfer	[145]
12	Bi_2_S_3_/SnIn_4_S_8_	heterojunction formation	microbial disinfection, RhB dye degradation	advanced redox ability	[146]
13	g-C_3_N_4_/Bi_2_WO_6_/AgI	Z-scheme	environmental decontamination	enhanced the separation and transfer of photoinduced charges	[147]
14	Bi_2_WO_6_/CdS	heterojunction formation	H_2_ evolution, RhB dye degradation	enhanced electrochemical properties	[148]
15	β-Bi_2_O_3_@g-C_3_N_4_	Z-scheme	tetracycline dye degradation	enhanced separation ability and prolonged lifespan of photogenerated charges	[149]

## Conclusion and Future Perspectives

There is great potential for Bi-based photocatalysts in the remediation of polluted environments and converting visible light into usable energy. In this study, we demonstrated that nanoscale Bi-based materials could be used for various photocatalytic ecological applications due to their distinctive electrical capabilities, crystal structure, and chemical attributes. These nanomaterials were obtained using various synthetic methods. Since there are constraints on employing just one Bi-based material as a photocatalyst, subsequent developments of Bi-based photocatalysts are explored in depth. The disadvantages are discussed in this article. We considered the most up-to-date research on Bi-based photocatalysts in our study. These developments stemmed from improvements in system design, microstructure management, and the synthesis of Bi-based composites. To further investigate the photocatalytic process, we analyzed the impact of several methods. The improved efficiency of photocatalysis based on Bi-valent cations was also covered.

Bi-based photocatalysts hold promise for environmental applications, but there is still much work to be done to improve the photocatalytic efficacy. Essential criteria in this field include the following: (1) Consideration must be given to the environmental and workplace safety and the economic impacts of the design process to overcome the “energy trilemma” when developing and building Bi-based photocatalysts. (2) The most difficult issue to address is obtaining and preserving the optimal nanostructure and morphology. (3) Thorough knowledge on reaction circumstances depending on structural traits is crucial. (4) Only there are only few studies on Bi-based photocatalysts in energy photocatalysis, such as H_2_ production, CO_2_ reduction, and selective organic transformation, because of the restricted number of photogenerated electrons from the less negative CB edge. (5) One of the most effective approaches to research reduction applications is to use Z-scheme heterojunctions with larger negative CB and photocatalytic Bi-based materials. (6) The stability of photocatalysts plays a vital role in large-scale application. Substantial research work has been already reported on it, but more ways to stabilize and recover a used Bi-based photocatalyst need to be found. Hence, more studies on the durability and renewability of Bi-based photocatalysts need to be carried out.
